# Evaluation of Brachial Plexus Using Combined Stereological Techniques of Diffusion Tensor Imaging and Fiber Tracking

**DOI:** 10.1055/s-0039-1687913

**Published:** 2019-06-12

**Authors:** Niyazi Acer, Mehmet Turgut

**Affiliations:** 1 Department of Anatomy, Erciyes University School of Medicine, Kayseri, Turkey; 2 Department of Neurosurgery, Adnan Menderes University School of Medicine, Aydın, Turkey; 3 Department of Histology and Embryology, Adnan Menderes University Health Sciences Institute, Aydın, Turkey

**Keywords:** axon number, brachial plexus, diffusion tensor imaging, stereology, tractography

## Abstract

**Background** Brachial plexus (BP) is composed of intercommunications among the ventral roots of the nerves C5, C6, C7, C8, and T1 in the neck. The in vivo and in vitro evaluation of axons of the peripheral nervous system is performed using different techniques. Recently, many studies describing the application of fiber tractography and stereological axon number estimation to peripheral nerves have been published.

**Methods** Various quantitative parameters of nerve fibers, including axon number, density, axonal area, and myelin thickness, can be estimated using stereological techniques. In vivo three-dimensional reconstruction of axons of BP can be visualized using a combined technique of diffusion tensor imaging (DTI) and fiber tracking with the potential to evaluate nerve fiber content.

**Conclusion** It is concluded that terminal branches of BP can be successfully visualized using DTI, which is a highly reproducible method for the evaluation of BP as it shows anatomical and functional features of neural structures. We believe that quantitative morphological findings obtained from BP will be useful for new experimental, developmental, and pathological studies in the future.

## Introduction

**Fig. 1:**
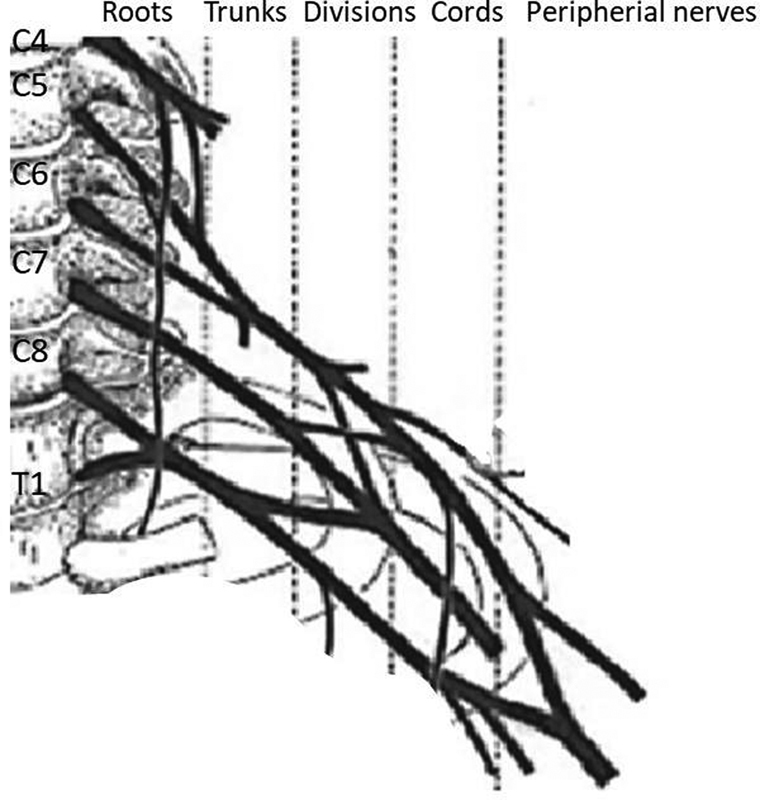
Schematic diagram showing the origins of the brachial plexus.

**Fig. 2:**
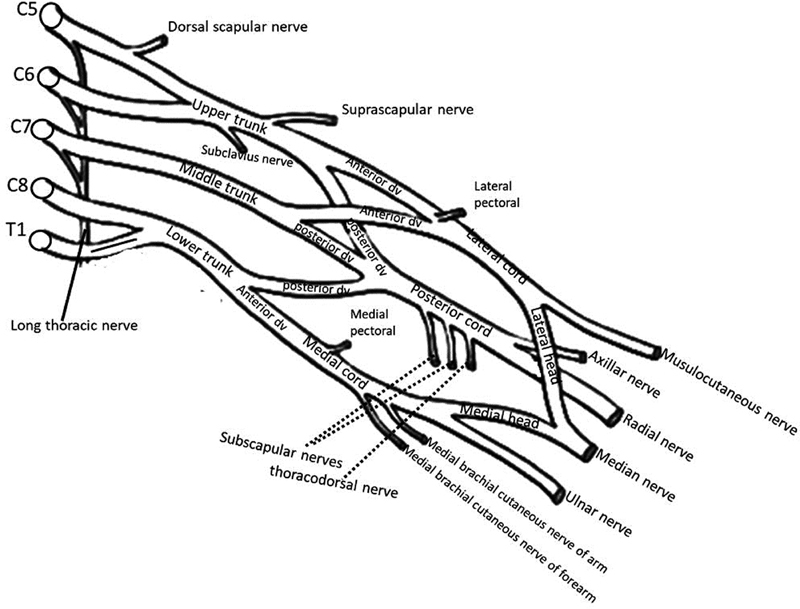
Terminal branches of the brachial plexus.

The brachial plexus (BP) has an important anatomical structure due to its close with the axillary region. 1 BP consists of ventral rami of spinal nerves C5, C6, C7, C8, and T1. 2 Anatomically, C5, C6, C7, C8, and T1 divide into ventral and dorsal rami. 3 Ventral rami of C5 and C6 join to comprise the “upper trunk,” C7 continues as the “middle trunk,” and C8 and T1 form the “lower trunk.” These trunks further subdivide into anterior and posterior divisions. 4 Anterior divisions of the upper and middle trunks fuse to comprise the “lateral cord,” anterior division of lower trunk continues as the “medial cord,” and posterior divisions of all three trunks comprise the “posterior cord.” 3 4 The major branches emanating out of these cords are axillary nerve, radial nerve, median nerve (MN), musculocutaneous nerve (MCN), upper and lower subscapular nerves, lateral pectoral nerve (LPN), medial pectoral nerve (MPN), and medial cutaneous nerve innervating arm, forearm, and pectoral region ( Figs. 1 and 2 ). 2 5

The ventral roots of spinal nerves (C5, C6, C7, C8, and T1) form BP. 6 C4 and T2 spinal nerves may contribute to the BP from variable level ( Fig. 1 ). 3 BP is composed of five anatomical parts; roots which are formed by the spinal nerves C5, C6, C7, C8 and T1, trunks, divisions, cords, and branches ( Fig. 1 ). 3 4 Trunks are divided into three parts: the superior, middle, and inferior. Each trunk divides into two branches: anterior and posterior. The suprascapular nerve and the subclavius nerve arise from the upper trunk. 3 4

The posterior cord is formed by the posterior branches of all trunks, whereas the lateral cord is formed by the anterior branches of superior and middle trunks; the anterior branch of the inferior trunk alone makes the medial cord ( Fig. 2 ). 1 2 3 The posterior cord of BP is formed by the union of the posterior divisions of the upper, middle, and lower trunks. The upper subscapular, thoracodorsal, lower subscapular, and axillary nerves arise from the posterior cord of BP and go further in the same direction distally as the radial nerve in the axilla ( Fig. 2 ). 3

The anterior divisions of the upper and middle trunks make the medial cord. The medial cord gives some branches: the MPN, ulnar nerve, and medial root of the MN ( Fig. 2 ). 6 7 The anterior division of the lower trunk makes the lateral cord of BP. 1 3 The LPN arises from the lateral cord as the first branch. 4 The remaining part of the lateral cord gives off MCN and lateral root of MN. 3 Therefore, MN is formed by the union of two roots: one from the lateral cord and the other from the medial cord. The lateral cord gives off the MCN ( Fig. 2 ). 3 4

Anatomical variations of BP are extremely common. 8 9 10 The existence of anatomic variations of the BP explains unexpected clinical signs and symptoms. 11 12 Variations in the formation of BP are important for performing surgical procedures in the neck, shoulder, and axillar regions. It is known that most of the human nerve lesions occur in the upper limb in the literature. 13 Jager et al 13 described an experimental crush injury model of the MN in a mouse.

High-resolution sonography has permitted appreciation of fine anatomical details of BP, such as the trunks, divisions, and cords, can be give us anatomical variability. 10 Furthermore, magnetic resonance imaging (MRI) can also be used to study the position of cords and trunks. 14

The morphology of nerve fibers is investigated by the peripheral nerves in various physiological and experimental conditions. 15 Terminal branches of the nerve fibers can give important information about axon growth and maturation. 16 Increased thickness of myelin sheath of a nerve fiber, in particular cross-sectional area (CSA) of axon, may be related with alterations in the ultrastructural features of the nerve fiber. 17

MRI scans can be acquired from live or postmortem specimens. They give a high-quality image and pronounced signal-to-noise ratio (SNR) since there are several advantages: they provide longer image acquisition times, and the images are not disrupted by motion and pulsation artifacts, providing higher resolution as well as improved SNR. Also, they provide evaluation of animal groups at the same age without any complications. In general, researchers are suspicious of MRI scans after death due to uncertainty regarding whether the fixation process changes the MRI measurements despite these advantages. 18

In a previous study, Oguz et al 18 conducted a volumetric analysis for a total of 22 neuroanatomical structures between in vivo and postmortem studies. They found no significant changes in volumetric measurements. 18 Lehmann et al 19 assessed the possible role of diffusion tensor imaging (DTI) to evaluate regeneration process in peripheral nerves, compared DTI parameters with morphometric measures using an experimental study of peripheral nerve regeneration in a mouse, and used DTI-based tractography to illustrate nerve regeneration. They compared histology and DTI after crushing of the sciatic nerve. 19 Different axonal diseases, including involvement of peripheral nerves with neoplastic disease or traumatic injury, lead to some changes in the structural integrity of the distal branches of BP, which can aid in both diagnosis and course of regeneration process following peripheral nerve injury.

In this article, we describe in detail how the axonal counts in the peripheral nerves were made using stereological analysis. Furthermore, the reproducibility of DTI tractography in healthy patients and in those with BP lesion will also be discussed in detail.

## Material and Methods

### Stereological Analysis of the Peripheral Nerves

#### Stereology

Stereology gives quantitative definitions of the geometry of three-dimensional (3D) structures from measurements using two-dimensional (2D) images for some biological materials for human or animals. 20 Stereological techniques can be used to obtain precise and unbiased estimations for both number and sizes of axons of the peripheral nerves. 21 22

Estimation of the total number and the absolute size distributions of both myelinated and unmyelinated axons using unbiased stereological techniques are described and evaluated in this study. 22 Axon numbers and areas are estimated using the fractionator, 2D nucleator, and point-counting technique. 17 21 A line grid is used to estimate the axon perimeter, and the direct orthogonal measurements in uniform, random locations is used to estimate myelin sheath thickness. 22

#### Stereological Estimation of Axon Number

##### Axon Number Estimation

Nerve specimens are fixed and then postfixed with some chemical substances: 2.5% glutaraldehyde in 0.1-M phosphate buffer for 4 to 6 hours in 4°C, postfix in 1% osmium tetroxide for 2 hours, dehydrate in an ascending alcohol series and embed in araldite. Series of semithin such as 1-μm-thick transverse sections are cut with ultramicrotome and stained with different histochemical substances. 21

##### True Fiber Number Counts

**Fig. 3:**
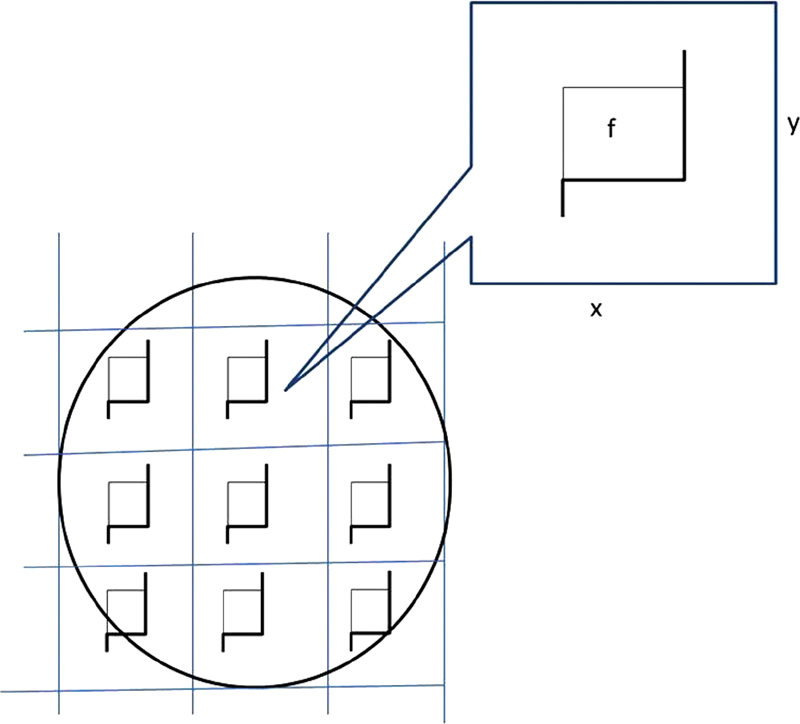
Nerve cross-section for the counting principle of the axon number. The section of nerve is sampled in a systematic random manner to gain an unbiased estimation of total axon number in a nerve. Each square represents a sampling area. An unbiased counting frame is seen in the center of this area. The axons are counted if nerve fiber is in the unbiased counting frame (f) in each sampling area. Estimates of the total number of myelinated axons are calculated as the product of the number of axons counted in a known fraction and multiplied by the inverse sampling fraction. Upper and right lines of unbiased counting frames represent the inclusion lines, whereas the lower and left lines including the extensions are the exclusion lines. Any profile of myelinated nerve fiber section hitting the exclusion lines is excluded, and profile of nerve fiber hitting the inclusion lines and located inside the frame is counted.

**Fig. 4:**
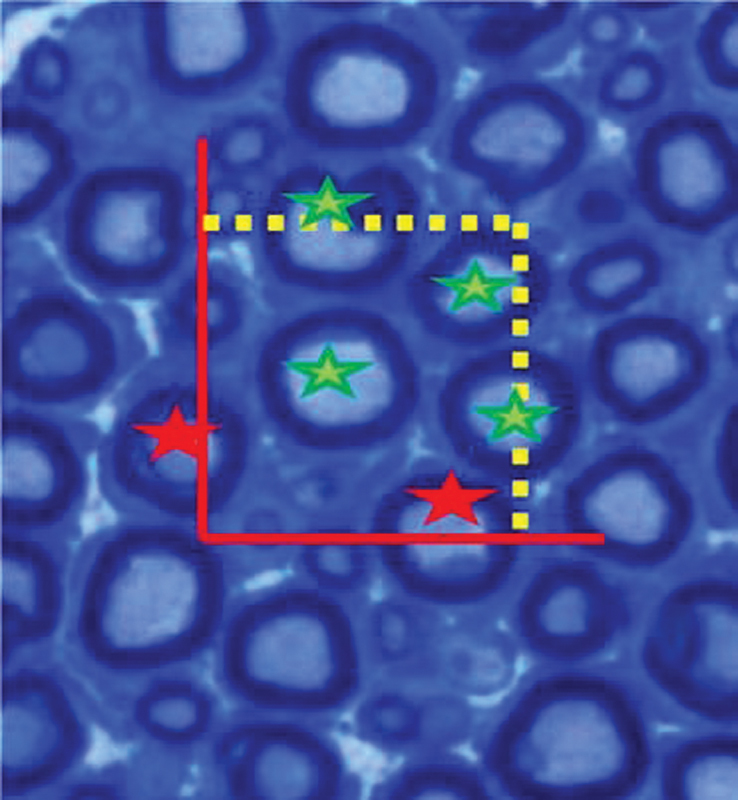
Unbiased counting frame method with a square sample area. The nerve profiles marked with *green stars* in panel A would be included in the counts but not the profiles marked with *red stars* .

The true number of myelinated or unmyelinated axons can be obtained using high-resolution light microscopy and compared as the first of choice standard for calibration of the stereological estimates. 23 One section from each nerve should be randomly selected and analyzed using a manual stereological workstation consisting of a digital camera and a light microscope. The area fraction method can be used to estimate all fibers count. 22 The area of step size and that of the microscopic counting frame must be equal ( Fig. 3 ). 17 Furthermore, to prevent counting twice the nerve fiber when intersecting two sequential microscopic fields, the unbiased counting frame method including inclusion and exclusion lines should be used; any profile of a myelinated nerve fiber touching the exclusion lines should be excluded from counting ( Fig. 4 ). 17

#### Stereological Estimates of Myelinated Axon Number

##### Unbiased Counting Frame

This method was originally described by Gundersen 24 and subsequently developed by Larsen 22 and Kaplan and coworkers 16 25 for application in stereology of peripheral nerve fiber. A square counting frame has two edges: one generally includes the inclusion and bottom lines and the other includes the exclusion and forbidden lines ( Fig. 4 ). It is excluded from counting when an axon touches these lines. 21 Inclusion lines are the upper and right edges of the frame ( Fig. 4 ), since an axon that is tangential to these lines is included for counting. 22 As a rule, an axon is thus excluded when an axon is tangential to both an inclusion line and an exclusion line. Profiles that are completely or partially within the frame are counted if they do not in any way touch any of neighboring frames below or to the left of the current frame demarcated by the exclusion lines and their infinite extensions. 21 Therefore, it is necessary to use counting frames, which are smaller than the whole printed area, to provide the examination of the “guard area” around each counting frame for confirmation that the extensions of a fiber profile that is partially within the frame and it is not tangential to any extension of the forbidden lines. 21

#### Total Number Estimation from Raw Stereological Data

The final total fiber number ( *N* ) is estimated using the following procedure: beginning with the raw data of axon number produced by different stereological protocols, *N* by multiplying the mean axonal density by the total CSA of the whole nerve measured at low magnification. 21 A fractionator approach is used, which does not necessitate the calculation of the total nerve area and thus prevents the variability arising from differences in the nerve area measurement. 21

In conclusion, by accepting the counting frame size (A _f_ ) and the step size (x-axis, y-axis) between frames (A _stp_ ), N was calculated by multiplying the number of fibers counted (ΣQ ^−^ ) by the sampling fraction area based on the following formula. 26



### Diffusion Tensor Imaging

MRI is a commonly used tool for noninvasive studies of the neural tissues. Modern MRI consists of various modalities such as including structural MRI, DTI, and functional MRI. 18 27 28 29 DTI is used to study the microstructural properties of white matter (WM) in the brain. 30 31 In recent years, DTI has been widely used for imaging of the peripheral nerves.

Nowadays, MRI is a well-known technique to study neural soft tissues, especially the brain. Also, MRI may provide invaluable information in various disorders of peripheral nerves due to compression, trauma, inflammation, or neuropathies. 32 3D course of WM tracts and orientation for the brain can be visualized with tractography. It is also possible to demonstrate peripheral nerve tracts in humans using DTI. 33 Diffusion tensor (DT) tractography can be done and provide for tracking peripheral nerves, and after peripheral nerve injury were determined to the relevance of the tracking parameters for evaluating fibers. 34 It was seen that DTI was useful in detecting any ischemic injury involving the optic nerve. 35

There are many diffusion parameters obtained from DTI data, such as individual eigenvalues (λ1, λ2, and λ3), longitudinal diffusivity (λ// = λ1), mean diffusivity (MD), radial diffusivity (RD; λ⊥= (λ2 + λ3)/2), and fractional anisotropy (FA), and generally used for quantitative characterization of nerve injuries. 27 28 29 36 37 The deviation of the diffusion ellipsoid from a spherical case describes FA. 31 38 39 The MD is a measure of the average molecular motion independent of any tissue directionality and proportional to the volume of the ellipsoid characterized by the DT. 39

Various diffusion indices derived from the DTI data, such as MD, RD, AD, and FA, are used for quantitative characterization of peripheral nerve. 19 40 In general, decreases in FA are associated with various WM neuropathologies including demyelination, ischemia, and inflammation. 41 42 FA describes the directional coherence of water diffision in tissue that is a quantitative parameter obtained from DTI, and is an indicator of the diameter and density of fibers, myelination process, and various structural characteristics. 41 43

After nerve injury and repair, DTI and fiber tracking can be used to track the progression of nerve regeneration. This may provide invaluable information in correct diagnosis of soft tissue tumors involving the peripheral nerves, nerve entrapment syndromes, and traumatic lesions of branches of the BP. 15

To measure tract density and length of the nerve can be made by placing a region of interest (ROI) over the structure being investigated using tractography-based methods. 27 28 29 44 Tractography provides the use of the principal direction of the tensor using individual streamlines from voxel-to-voxel after the tensor direction like reconstruct the WM tract. 45 Analyses of fiber tracts help combine these streamlines for the detection of WM and peripheral nerve for ROIs (e.g., corpus callosum, MN: median nerve). 46

### Data Processing

DT fitting is performed using different software such as DTIStudio and FiberTrak. 45 47 Fiber tractography is performed using a multiple-seed ROI technique and employs a fiber assignment using the continuous tracking (FACT) method. 45 48 Using this method, the tensor was diagonalized to produce three eigenvalues (λ1– λ3) and corresponding eigenvectors (ν1–ν3). 37

The DTI datasets were transferred to a personal computer with Windows platform and processed using the analysis software DTIStudio developed and distributed by H. Jiang and S. Mori, Johns Hopkins University and Kennedy Krieger Institute ( http://godzilla.kennedykrieger.org or http://lbam.med.jhmi.edu ). 45 To eliminate any potential small bulk motions encountered during the scans, images were realigned using the AIR program. Subsequently, when subject motion and instrumental malfunction are seen, all diffusion-weighted images must be visually inspected for apparent artifacts. Using the multivariate linear fitting, the six elements of the DT were calculated for each pixel. 29 The optimal value for the number of directions can be used in nervous system tractography (a total of 32 directions). 40 49 Using DT tractography, the fiber counts of terminal branches of BP can be calculated.

### Fiber Tracking and ROI Drawing Strategy

The fiber assignment by continuous tracking or FACT method for 3D tract reconstruction is usually performed with an FA threshold of 0.2 and an inner product threshold of 0.75, which is larger than 60 degrees prohibited angles during tracking to reconstruct tracts of interest that is exploits current anatomical knowledge of tract trajectories, using a multi-ROI approach. 31 50 There are three types of operations, namely “AND,” “CUT,” and “NOT,” when multiple ROIs are used for a tract of interest. 51 The fiber tracking can start and stop when the FA value is above and below 0.2 or when the angular deviation can be larger or shorter than 60 degrees. 31 50

**Fig. 5:**
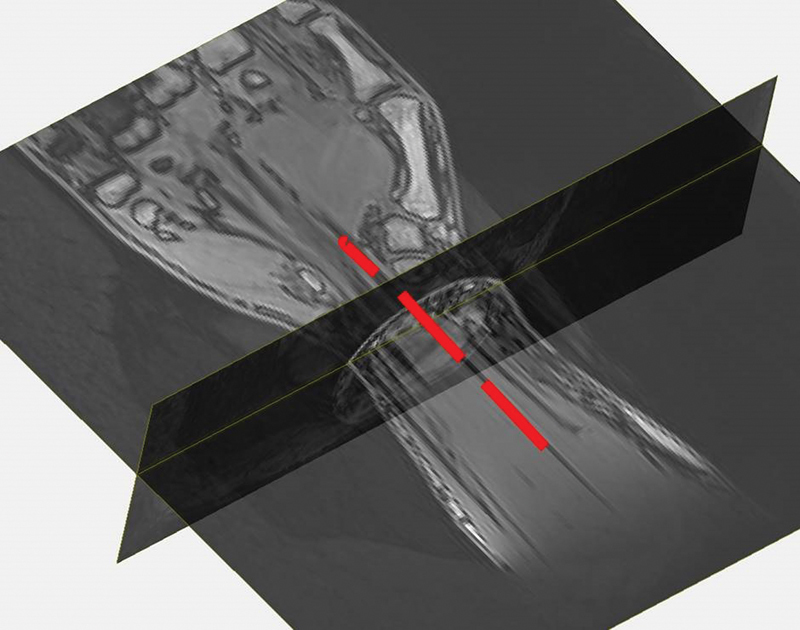
Three-dimensional image illustrates automatic fiber tracking of the median nerve.

**Fig. 6:**
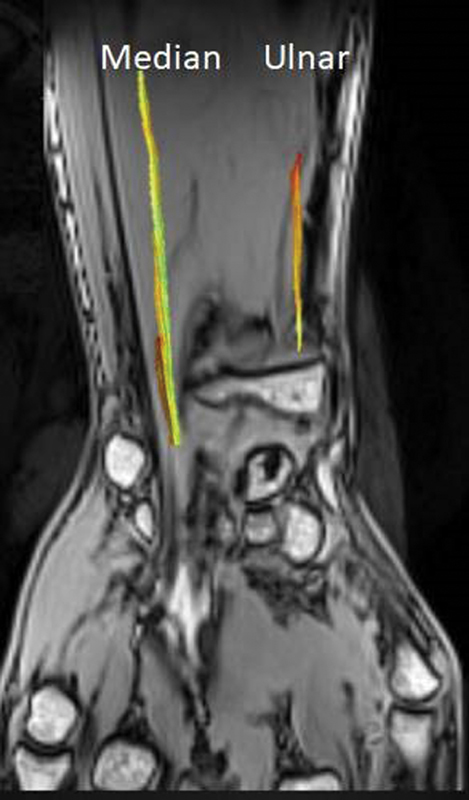
Three-dimensional image illustrates fiber tracking of the median nerve and ulnar nerve.

Recently, there are several studies on the application of DTI and fiber tractography of peripheral nerves, and most of them are related to MN and ulnar nerve and have been published in the literature ( Figs. 5 and 6 ). 15 36 37 The accuracy of DT measurements with echo-planar imaging is restricted by the low- resolution image; therefore, tractography of peripheral nerves is insufficient for the identification and visualization of bundles of nerve fibers owing to the low SNR in DTI of the peripheral nerve.

Technically, DTI scans with increased resolution are used to obtain detailed information about peripheral nerves; thus, both visual data and correct mathematical values such as fiber count for peripheral nerves including the MN can be produced in detail.

**Fig. 7:**
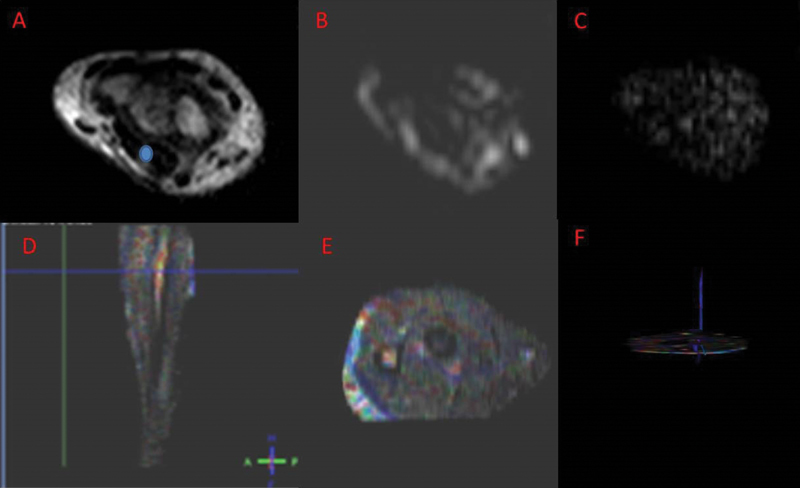
Right (peripheral nerve schwannoma) wrists of a 27-year-old female. T1-weighted magnetic resonance imaging (MRI; **A** ) at the level of the elbow shows the right median nerve (MN; region of interest). Corresponding ( **B** ) diffusion-weighted imaging and ( **C** ) diffusion tensor imaging maps. ( **D** ) Proximal level of the forearm . ( **E** ) ColorMap. ( **F** ) MN of the forearm. Tractography images show the MN in *blue color* .

Recently, we performed tractography (3D fiber tracking) using the 1.5 or 3 Tesla MRI scanner and reconstructed MN using DTI assessment because it may serve in the diagnosis of peripheral nerve schwannoma ( Fig. 7 ).

## Discussion

The anatomical variations of BP are well studied for cadaver and living subjects including human infant and adult using dissection technique, ultrasonography, and MRI. 9 10 DTI allowed visualization of BP using tractography. Recent studies have revealed an increased interest in this technique for peripheral nerve anatomy and lesions such as carpal tunnel syndrome (CTS). 47 Some studies measured FA, MD, and apparent diffusion coefficient (ADC) of BP in healthy and BP injury participants at 1.5 and 3 T. 51 52 53 Ho et al 53 measured FA and MD for BP and found no significant difference in intraclass correlation coefficients for inter and intrareader agreement. Kabakci et al 54 found a decrease in FA value and an increase in ADC with advancing age. Moreover, Barcelo et al 55 observed a significant difference in FA values between healthy subjects and patients with CTS. Stein et al 56 also found that FA, RD, and ADC differed statistically between healthy subjects and patients with CTS. According to this study, in healthy subjects, the FA increased and the RD and ADC decreased within the carpal tunnel regions. 56 More recently, Oudeman et al 57 measured FA, MD, AD, and RD for the individual roots and trunks in a total of 30 healthy volunteers using DT-MRI of BP. They found no significant difference between sides and root levels. 57

In 2013, Chhabra et al 58 examined a total of 29 patients with neoplastic lesions and tumorlike conditions using 3T MRI with anatomical and functional diffusion, diffusion-weighted imaging (DWI), and DTI techniques. In their study, they evaluated ADC of the lesion and FA of nerves with interobserver reliability in measurements of ADC and FA. 58 Importantly, they did not find any significant difference in FA or ADC between men and women. 58 More importantly, however, they found that the FA of involved nerves was lower than that of contralateral healthy nerves. 58 Moreover, they reported ADC on DTI to be more useful than DWI in differentiating benign from malignant lesions, suggesting DTI as a reliable method in the noninvasive evaluation of neoplastic diseases involving the peripheral nerves. 58

Today, it is widely accepted that the use of DTI and tractography may provide a differential diagnosis in patients with benign and malignant tumors involving the peripheral nerves. 59 It has been suggested that the malignant tumors of the nerves have rapid tumor growth and that the fibers are destroyed as a result of anatomical disruption possibly owing to malignancy. 58 Therefore, it is possible that DTI may provide further information for uncovering of the underlying pathophysiological mechanism and surgical treatment of patients with tumors involving the terminal branches of the BP.

## Conclusion

Clinically, use of the BP in experimental research, tractography, and axonal count studies may be more relevant because the most of peripheral nerve injuries in humans involve the upper extremity. Axon number estimation using stereology for terminal braches for BP can be performed unbiasedly. DTI provides some information about the terminal branches of BP for 3D application to living humans. In peripheral nerves such as MN, derived FA, MD, RD, and fiber count can be calculated using DTI. In clinical practice, fiber tracking may offer a new approach for imaging of diseases of BP and help in the assessment of neoplastic pathologies involving the terminal branches of BP.
